# Pattern electroretinogram (PERG) in the early diagnosis of normal-tension preperimetric glaucoma: a case report

**DOI:** 10.1007/s10633-013-9414-x

**Published:** 2013-10-19

**Authors:** Joanna Karaśkiewicz, Monika Drobek-Słowik, Wojciech Lubiński

**Affiliations:** Department of Ophthalmology, Pomeranian Medical University, Powstańców Wlkp. 72, 70-111 Szczecin, Poland

**Keywords:** Preperimetric normal-tension glaucoma, PERG

## Abstract

**Introduction:**

The purpose of this case is to present the use of pattern electroretinogram (PERG) in the early diagnosis of normal-tension preperimetric glaucoma in 56 years old woman.

**Methods and Results:**

At baseline the results were as follows: distance-corrected visual acuity in the right eye (RE) and left eye (LE) 1.0 and 0.7, respectively (Snellen table), normal anterior segments in both eyes, normal fundus in the RE and abnormal cup to disc ratio (0.6) in the LE. Intraocular pressure (IOP) was within normal limits in both eyes: RE-14 mmHg, LE-18 mmHg (Goldmann tonometer). Results of standard automated perimetry (SAP), short wavelength automated perimetry (SWAP) and nerve fiber analyzer (GDx) were normal in both eyes. PERG result was normal in the RE but in the LE reduced amplitudes of P50 and N95 waves were observed. After topical treatment (Xalacom to the LE), a reduction of IOP to 13 mmHg was achieved and was accompanied by amplitudes increase of PERG waves. After discontinuation of the therapy, IOP increased to 18 mmHg and P50 and N95 amplitudes decreased to the values before treatment, suggesting the influence of IOP lowering therapy on electrical function of retinal ganglion cells. After 4 years from the baseline, static perimetry results were still normal, but abnormalities in retinal nerve fiber layer thickness were detected in GDx.

**Conclusions:**

PERG was a useful test not only for the early diagnosis of normal-tension preperimetric glaucoma, but also in evaluating the effectiveness of antiglaucomatous treatment.

## Introduction

Normal-tension glaucoma is defined as a primary open-angle glaucoma (POAG), where all signs of the disease are present, except from the elevated intraocular pressure (IOP). Normal-tension POAG is diagnosed on the basis of the optic nerve head damage typical for glaucoma, open anterior chamber angle in gonioscopy, glaucomatous visual field (VF) defects, and normal IOP, which is not higher than 21 mmHg without treatment [1]. Unfortunately, there are no characteristic abnormalities of either VF or optic disc that can be diagnostic for the normal-tension glaucoma [2]. Although the pathomechanism of normal-tension glaucoma is still unknown, there are some features that may occur more often with this disease: female sex, migraine headache, Raynauld phenomenon, ischemic vascular diseases, autoimmune diseases, and coexistence of macro- and microvascular abnormalities or coagulopathies [2, 3]. The diagnosis of glaucoma becomes more complicated when neither VF, nor nerve fiber layer shows any pathological changes. The individual-based decision to start therapy or to wait and observe without treatment may depend on further investigations and should be supported by additional data, which will allow to measure the function of retinal ganglion cells (RGCs) that are damaged or impaired in glaucoma. An example of such an examination is a commonly used and well-known pattern electroretinogram (PERG), performed according to the International Society for Clinical Electrophysiology of Vision (ISCEV) [4].

Transient PERG was recorded with the RetiPort (Roland Consult Instr.) system. Protocol of the PERG test was implemented in the original software of the system. Patient’s pupils were not dilated, monocular stimulation was used, refraction correction was applied with respect to the eye-screen distance 0.5 m, and interruptions of the test were introduced when frequent blinking or fixation loose was observed (patient was monitored with a TV camera).

Parameters of the pattern stimulation were as follows: 21″ CRT monitor with a frame rate equal to 75 fps was used; black and white reversing checkerboard (30° FOV) was presented to the patient, with a check size equal to 1°2′; luminance for white elements: 120 cd/m^2^, contrast: 97 %; temporal frequency was equal to 4.6 rps (2.3 Hz); and central fixation was used.

### Electrodes

Thread DTL electrode was used as active, and gold disc was placed at the ipsilateral outer canthus as reference, with ground (gold disc) electrode placed on the forehead (Fpz).

Parameters of the recording system were as follows: amplifiers sensitivity: 20 μV/div, filters: 1–100 Hz. Notch filters: off. Artifact reject threshold: 95 % (for the amplifiers range ± 100 μV). Sweep time: 250 ms (time base: 25 ms/div). Averaging: 200 sweeps. Two consecutive waveforms were recorded, off-line averaged, and then analyzed.

### Results analysis

According to the standard, amplitude as well as time parameters of the obtained waveforms were analyzed; manual correction was applied to the automatic cursors placement. Values of all parameters were compared with the own laboratory normal values.

In this study, we describe a case, where PERG strongly suggested a recognition of normal-tension preperimetric glaucoma 4 years before it was finally diagnosed, according to the actual criteria: a glaucomatous appearance of the optic disc head, open chamber angle, and thinning of retinal nerve fiber layer (RNFL) thickness measured by GDx or optical coherence tomography (OCT), with normal VF, confirmed by at least two following standard automated perimetry (SAP) examinations [5].

## Case

**Fig. 1:**
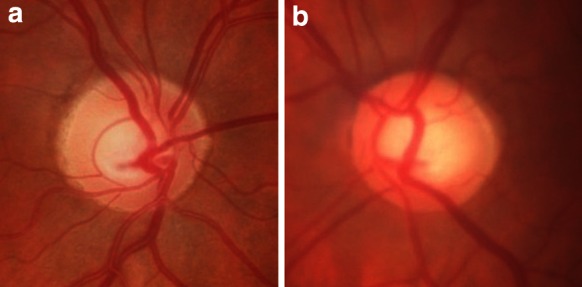
Fundus photographs of the *right* (**a**) and *left* (**b**) eyes. Increased cup-to-disc ratio (0, 6) in the left eye was detected

**Fig. 2:**
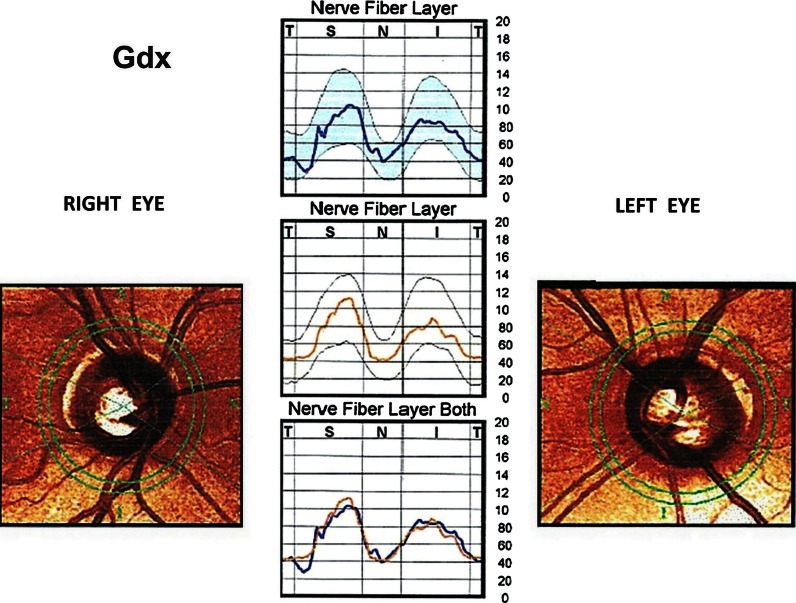
Gdx results in both eyes within normal limits

**Fig. 3:**
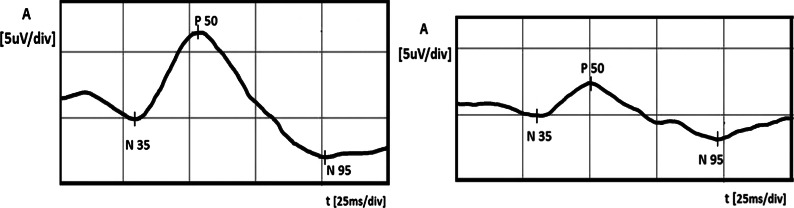
Right eye (*left side*): PERG amplitudes of P50 and N95 waves within normal limits. Left eye (*right side*): lower PERG amplitudes of P50 and N95 waves

A 56-year-old healthy white female was examined by an ophthalmologist, because her mother suffered from glaucoma in both eyes and she wanted to check herself. In a routine ophthalmological examination at baseline, patient’s distance-corrected visual acuity (DCVA) was 1.0 in the right eye (RE) and 0.7 in the left eye (LE) (Snellen table). IOP was 14 mmHg and 18 mmHg in the right and left eyes, respectively (Goldmann tonometer). The anterior segments were normal in both eyes including open anterior chamber in gonioscopy. Stereoscopic fundus examination result was normal in the RE and the only abnormality in the LE was an increased cup-to-disc ratio (c/d ratio 0.6) (Fig. 1), what was confirmed by Heidelberg retinal tomography (HRT). Pachymetry results were normal and equal to 517 and 521 μm in the RE and LE, respectively. In both eyes, SAP, short wavelength automated perimetry (SWAP) results, and RNFL thickness measured by GDx were as well within normal limits (Fig. 2). Additionally, PERG test was performed and the results were as follows: RE—normal amplitude (A) of P50 (6.8 μV) and A N95 (9.6 μV) waves, LE—reduced amplitudes of P50 (2.4 μV) and N95 (4.1 μV) waves. Implicit time of the RE and LE was normal and equal to 53.6 and 50.9 ms, respectively (Fig. 3). In our laboratory, the established normal values (mean ± 2SD) for this patient’s age are as follows: A P50: 3.2–11.3 μV; IT P50: 46.5–59.2 ms; A N95: 4.8–15.7 μV. According to the well-known guidelines, the coefficient of variation (CV) of amplitude for the transient PERG is 9 ± 1 % [6] and we assumed, according to the study presented by other authors [7], that an increase or decrease in PERG amplitude is diagnosed when it exceeds 20 %.

**Fig. 4:**
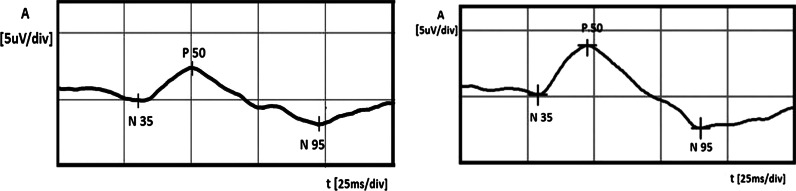
Left eye: increase in PERG amplitudes after instillation of Xalacom (*right side*), in comparison with the score before treatment (*left side*)

**Fig. 5:**
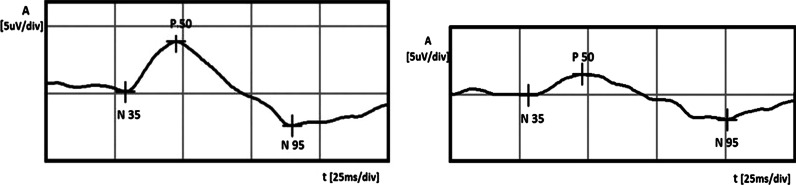
Left eye: decrease in PERG amplitudes after 1-month discontinuation of Xalacom therapy (*left side* treated eye, *right side* without treatment)

In this case, in the LE, normal-tension preperimetric glaucoma was suspected, because of the repeatable abnormal PERG results, increased c/d ratio in the LE, and higher IOP in the LE in comparison with the RE. We decided the instillation one drop of Xalatan (Latanoprost) to the LE once a day, but because after 1 month, IOP reduction was less than 25 % (from 18 to 16 mmHg, 11 % decrease) and the treatment was changed to Xalacom (Latanoprost + Timolol) in the same regiment. After 3 months from the beginning of the therapy, the IOP in the LE decreased up to 13 mmHg (28 % fall from the baseline) and the patient was referred to the electrophysiological laboratory to repeat the PERG. The values of the amplitudes in the LE much improved [A P50 from 2.4 to 3.8 μV (58 % growth, CV = 32 %), A N95 from 4.1 to 6.3 μV (54 % growth, CV = 30 %)] and suggested the improvement of RGCs function (Fig. 4). After the above-mentioned treatment, the amplitudes achieved values near lower limits of normal. Implicit time values did not show any changes. For checking that IOP-lowering therapy is the cause of RGCs’ function improvement, we decided to stop Xalacom instillation for 1 month. After this time period, IOP in the LE increased to the initial value (18 mmHg), and in the control, PERG A of P50 and N95 waves again decreased to the following values: A P50: from 3.8 to 1.5 μV (60 % decrease, CV = 61 %), A N95: from 6.3 to 3.4 μV (46 % decrease, CV = 42 %) (Fig. 5). Implicit time values did not show any changes. The decision to include Xalacom to the LE as a permanent therapy was made.

At the follow-up, 8 months later, the IOP in the LE was equal to 12 mmHg and to check again RGSc’ function, the patient was referred to the electrophysiological laboratory to repeat the PERG. In this examination, both amplitudes did not differ much with the values at baseline: A P50 (3 % fall, CV = 2 %) and A N95 (8 % growth, CV = 6 %). IT values again did not show any changes.

**Fig. 6:**
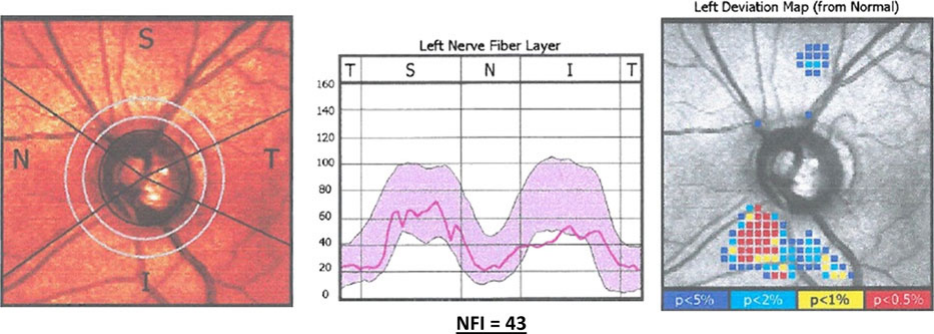
Left eye: gdx result after 4 years of follow-up: abnormal thickness of RNFL in the lower quadrant and a pathological nerve fiber index (NFI) = 43

Once a year, in our patient, the following examinations were performed: SAP, SWAP, Gdx, and PERG. No considerable changes with the previous results were detected in above-mentioned tests. After 4 years from the initial examination, static perimetry tests were repeated. Both SAP and SWAP results were still normal in the LE. Additionally, to find out whether the reduced function of RGCs in the LE corresponds with the abnormalities in RNFL, Gdx was performed (Fig. 6). Its results revealed abnormal thickness of RNFL in the lower quadrant and a pathological nerve fiber index (NFI), which was 43 in the LE. At this point, the normal-tension preperimetric glaucoma was decisively diagnosed in the LE, because it was in an agreement with a current definition of this disease.

## Discussion

Normal-tension preperimetric glaucoma may be difficult to diagnose for the ophthalmologist and is complicated for the patient, who may have difficulties with understanding that he really suffers from glaucoma, even though his IOP and VFs are normal. To diagnose, very often, additional glaucomatous tests are needed. In our case report, apart from commonly used tests, PERG was performed. It is known from the literature that PERG may be an early glaucoma indicator in either OHT patients, or glaucoma suspects [8–10]. The results of these surveys showed that PERG helped to identify glaucoma-suspected patients, whose perimetry results were normal [9] or who had OHT and were at a high risk to develop glaucoma in the future [8, 10, 11]. Additionally, it is documented that PERG can measure the influence of IOP-lowering treatment on RGCs function in early glaucoma [12] and glaucoma suspects [13]. These promising results were the reason, why in our difficult diagnostic patient, PERG was registered.

In the presented case, initial results of commonly used tests like Gdx, SAP, and SWAP were normal in both eyes. The reasons to start pharmacological IOP-lowering therapy in the LE were as follows: family glaucomatous history, asymmetry of IOP between eyes (in the LE 4 mmHg higher than in the RE), increased c/d ratio (0.6) in the LE confirmed by HRT, lack of other systemic and eye diseases that could destroy an electrical function of RGCs, and finally, reduced PERG amplitudes of P50 and N95 waves at baseline, only in the LE. Nowadays, *European Glaucoma Society (EGS)* recommends 25 % decrease in IOP from baseline [1] to reduce the risk of progression by 50 % in cases of early glaucoma patients. Because after instillation of Xalatan (Latanoprost), IOP was reduced less than 25 % and Xalacom (Bimatoprost + Timolol) was prescribed. This drug is known to meet the criteria of EGS connected with an appropriate lowering IOP [14].

After 3 months of Xalacom therapy, IOP decreased (28 %) what was accompanied with PERG amplitudes increase. It suggested that at the time of the treatment’s initiation, glaucoma was at its early stage, and therefore, some RGCs were just dysfunctional and so their partial function’s improvement after IOP-lowering therapy was possible and detected by PERG. Such an improvement was described also by Ventura et al. [[15], [13]]. In these studies, instillation of IOP-lowering therapy in early glaucoma patients was also a cause of RGCs function improvement. In the presented case, increased IOP and associated decreased PERG amplitudes after 1-month discontinuation of Xalacom confirmed that IOP-lowering therapy was the cause of RGCs’ recovery.

This study’s results also show that even properly treated normal-tension glaucoma, which according to the *collaborative normal*-*tension glaucoma study group* means decreased IOP of 30 % from the baseline [16], does not have to stop the impairment of following RGCs. In the case of this patient, even though the IOP reduction near 30 % was obtained, after 8 months of treatment, the PERG amplitudes were not higher, but did not differ much with the initial values, what was another indicator of progressive destruction of RGCs, typical for glaucoma. The fact that glaucoma is a progressive disease, despite the drug therapy, is well known, especially in the normal-tension glaucoma, caused by multifocal components, where IOP-lowering treatment cannot stop the progression either [16]. The consequences of progressive loss of RGCs were the characteristic features observed in Gdx after 4 years from the baseline, manifested by nerve fiber loss. Such a sequence of events is consistent with the well-known information in the literature that PERG can detect RGCs dysfunction that occurs before cell death [18] and the changes of RNFL thickness are found in structural tests like Gdx before VF loss detected by SAP [19].

In conclusion, PERG was a useful test for the early diagnosis of normal-tension preperimetric glaucoma before the changes in other studies were observed. Its results enabled not only to start antiglaucomatous treatment 4 years before the diagnosis according to the actual criteria, but also to evaluate the effectiveness of the therapy. What is the most important, early inclusion of the treatment reducing IOP, with a high probability, slowed down a progression of glaucomatous optic neuropathy in this case.
